# Precursor A-Kinase Anchor Protein 4 as a Predictive Biomarker of Post-Thaw Semen Quality in Goats

**DOI:** 10.3390/vetsci12101003

**Published:** 2025-10-16

**Authors:** Ahmet Eser, Abdurrahman Alakuş, Kemal Bağcı, Aslıhan Çakır Cihangiroğlu, Selin Yağcıoğlu, Ramazan Arıcı, Kamber Demir

**Affiliations:** 1 Department of Reproduction and Artificial Insemination, Faculty of Veterinary Medicine, Siirt University, Siirt TR-56100, Türkiye; abdurrahman.alakus@siirt.edu.tr (A.A.); kemal.bagci@siirt.edu.tr (K.B.); aslihan.cakir@siirt.edu.tr (A.Ç.C.); 2 Department of Reproduction and Artificial Insemination, Faculty of Veterinary Medicine, Istanbul University-Cerrahpaşa, Istanbul TR-34320, Türkiye; selin.yagcioglu@iuc.edu.tr (S.Y.); ramazan.arici@iuc.edu.tr (R.A.); kamberdemir@iuc.edu.tr (K.D.)

**Keywords:** proAKAP4, goat semen, cryopreservation, sperm quality, biomarker, flow cytometry

## Abstract

**Simple Summary:**

Freezing and thawing semen are commonly used in animal breeding but often reduce sperm quality, which can affect fertility. Scientists are looking for simple ways to predict semen quality after thawing so that only the best samples are used for reproduction. In this study, we examined a protein found in sperm called precursor A-kinase anchor protein 4. We wanted to see whether the level of this protein could tell us something about how healthy the sperm would be after thawing. Semen was collected from goats, frozen, and then thawed. We measured the amount of this protein and compared it with many features of sperm, such as their ability to move, stay alive, and keep their cell structures intact. We found that sperm samples with higher amounts of the protein showed better movement, higher survival, stronger cell membranes, and more active energy production. However, one structure of the sperm, called the acrosome, was not affected. These results suggest that measuring this protein can help identify high-quality semen samples after freezing and thawing, which may improve breeding success and benefit animal production.

**Abstract:**

The evaluation of sperm proteins has emerged as a promising approach to predicting semen quality across animal species. This study investigated the relationship between post-thaw concentrations of precursor A-kinase anchor protein 4 (proAKAP4) and objective sperm quality parameters in goats. Semen was collected from 16 adult goats (Boer, *n* = 8; Anglo-Nubian, *n* = 8) and frozen using a standardized protocol with OptiXcell (IMV Technologies, l′Aigle, France) extender (*n* = 5). After thawing, proAKAP4 concentrations were measured with an enzyme-linked immunosorbent assay (ELISA), while sperm motility and kinematics were assessed with computer-assisted analysis (CASA), and viability, plasma membrane integrity, acrosome integrity, and mitochondrial activity were evaluated using flow cytometry. Samples were grouped according to low, medium, or high proAKAP4 levels for comparison, and correlations with sperm parameters were examined. The results showed that semen with higher proAKAP4 concentrations had significantly greater total and progressive motility, more favorable kinematic values, and improved viability, plasma membrane integrity, and mitochondrial function (*p* < 0.05), whereas acrosome integrity was not influenced (*p* > 0.05). The average post-thaw proAKAP4 concentration was 38.66 ± 1.11 ng/10^6^ sperm, and no differences were observed between Boer and Anglo-Nubian breeds (*p* > 0.05). These findings indicate that proAKAP4 is strongly associated with multiple sperm functional traits and may serve as a reliable biomarker for assessing post-thaw semen quality in goats.

## 1. Introduction

The success of sperm cryopreservation is variable and influenced by numerous biological and technical factors. Post-thaw sperm quality is paramount for ensuring effective fertilization. Various parameters can affect post-thaw viability and functionality, including the choice of cryoprotectants, freezing techniques, sperm dilution rates, and the inherent biological characteristics of sperm [1,2,3,4].

Semen analysis remains fundamental for evaluating and predicting post-thaw sperm viability. Advanced techniques such as Computer-Assisted Sperm Analysis (CASA) provide precise quantification of motility parameters, which serve as critical indicators of functional competence [5]. In addition, fluorescence-based assays and flow cytometric analyses represent powerful methodologies for assessing sperm viability, functionality, and overall reproductive potential, particularly in the context of cryopreservation. These techniques utilize fluorescent probes to interact selectively with various components of sperm cells, enabling the quantification and characterization of cellular properties essential for fertility, such as plasma membrane integrity, mitochondrial functionality, and deoxyribonucleic acid (DNA) integrity [6,7]. Moreover, fluorescent dyes can be used to label distinct cellular structures or indicate functional states, making flow cytometry an indispensable tool for evaluating sperm quality in both fresh and cryopreserved samples [7].

With the advancement of technology, the integration of analytical methodologies such as proteomics has provided deeper insights into protein expression patterns and metabolic alterations during cryopreservation. These approaches not only improve the understanding of sperm physiology but also facilitate the identification of potential biomarkers for predicting post-thaw sperm quality [8]. The interplay between sperm proteins, biomarkers, and conventional or advanced spermatological analyses provides an essential foundation for advancing reproductive science. Understanding the biochemical landscape of sperm and relating these factors to fertility outcomes not only emphasize the importance of precise sperm assessments but also pave the way for future research to enhance reproductive technologies and optimize outcomes in fertility treatments. As methodologies continue to evolve, the integration of molecular biology, proteomics, and advanced analyses will yield further insights into the complex dynamics of male fertility. Approximately 5000 proteins in semen are estimated to be associated with male fertility potential [9,10]. However, since a significant portion of these proteins originates from seminal plasma, their presence and abundance are highly sensitive to various external factors, including nutritional status and ejaculation frequency. Additionally, the semen dilution rate poses a challenge in detecting seminal plasma protein biomarkers in frozen-thawed semen [11,12,13,14]. For this reason, biomarkers located within the sperm structure are considered more reliable than those derived from seminal plasma for estimating the potential fertility of sperm [15].

The objective of this study was to investigate the relationship between precursor A-kinase anchor protein 4 (proAKAP4) levels and post-thaw semen quality parameters in Boer and Anglo-Nubian goat spermatozoa. ProAKAP4 is a sperm-specific protein that has emerged as a promising biomarker for semen quality assessment in various mammalian species [15,16,17,18]. However, studies in small ruminants [19], particularly in goats [20], remain limited. Moreover, previous investigations in goat semen have not utilized comprehensive objective evaluation methodologies such as flow cytometry. Therefore, this study aimed to: (1) evaluate proAKAP4 protein concentrations in cryopreserved semen samples from Boer and Anglo-Nubian goats, (2) assess post-thaw sperm quality parameters using objective analytical methods including CASA and flow cytometry, and (3) determine the correlations between proAKAP4 levels and various spermatological parameters to establish its potential as a reliable biomarker for semen quality evaluation in caprine species.

## 2. Materials and Methods

### 2.1. Animals, Semen Collection and Initial Evaluation

In this study, a total of 16 healthy male goats from two breeds—Boer (*n* = 8) and Anglo-Nubian (*n* = 8)—aged between 2 and 3 years, were included. All experimental procedures were conducted at the Small Ruminant Reproductive Biotechnology Research Center, Siirt, Türkiye (37°55′30″ N, 41°56′45″ E; 895 m above sea level). The animals were housed under uniform management conditions, with free access to clean water and natural daylight. All goats were fed a balanced diet consisting of alfalfa hay and a commercial concentrate ration formulated to meet the nutritional requirements for maintenance and reproduction.

All procedures involving animals were performed in accordance with the ethical principles of animal experimentation and approved by the Local Ethics Committee for Animal Research at Siirt University, Türkiye (20 April 2025).

Semen samples were collected from each goat outside the breeding season. Collection was performed once per week using an electroejaculator (e320, Minitube, Tiefenbach, Germany), following the procedure described by Ungerfeld et al. [21], with a total of five ejaculates obtained from each goat (*n* = 5). Collected samples were immediately placed in a water bath maintained at 26 °C. Ejaculate volume was measured using a graduated conical tube with 0.1 mL scale intervals, and sperm concentration (per mL) was determined using a photometric method (Ovine-Caprine AccuRead, IMV Technologies, l′Aigle, France). Semen mass motility was assessed under a phase-contrast microscope (Eclipse Ci-L, Nikon, Tokyo, Japan) at 5× magnification, evaluating swirling movement at the periphery of a drop of semen and scoring motility on a 4-point scale according to the vigor of the wave-like motion. Individual sperm motility was assessed using computer-assisted sperm analysis (CASA). In this study, only ejaculates with a sperm concentration ≥1.0 × 10^9^ sperm/mL, mass motility ≥+3, and subjective progressive motility ≥75% were selected for cryopreservation [22].

### 2.2. Semen Cryopreservation

A total of 80 ejaculates (Boer, *n* = 40; Anglo-Nubian, *n* = 40) were diluted with a commercial extender (OptiXcell^®^, IMV Technologies, l′Aigle, France). The dilution ratio was adjusted to obtain 100 × 10^6^ motile spermatozoa per 0.25 mL mini straw. Dilution was performed gradually at 26 °C. Subsequently, the samples were cooled to 5 °C and equilibrated for 2 h. Semen loading into straws (MPP Uno, Minitube, Tiefenbach, Germany) and freezing were carried out using automated systems (Icecube 14S, Sylab, Purkersdorf, Austria). The freezing protocol consisted of cooling from 5 °C to −8 °C at a rate of 3 °C/min, followed by cooling from −8 °C to −120 °C at a rate of 15 °C/min [23]. After freezing, samples were stored in liquid nitrogen (−196 °C).

### 2.3. Post-Thaw Spermatological Analyses

For post-thaw analysis, two straws per individual were thawed at 37 °C for 30 s. The samples were then pooled and evaluated for motility and kinematic parameters using CASA. In addition, plasma membrane integrity, acrosome integrity, viability, and mitochondrial membrane potential were assessed using flow cytometry. The proAKAP4 concentration in post-thaw semen samples was measured by enzyme-linked immunosorbent assay (ELISA).

#### 2.3.1. Evaluation of Post-Thaw Semen Motility and Kinematics

Total motility (MOT, %), progressive motility (pMOT, %), and kinematic parameters—including mean path velocity (VAP, µm/s), straight-line velocity (VSL, µm/s), curvilinear velocity (VCL, µm/s), amplitude of lateral head displacement (ALH, µm), beat cross frequency (BCF, Hz), straightness (STR = VSL/VAP), linearity (LIN = VSL/VCL, %), and wobble (WOB = VAP/VCL, %)—were assessed using a CASA system calibrated for goat sperm (SCA, Microptics, S.L., Version 3.2.0, Barcelona, Spain) equipped with a Basler ACA1300-200UC camera (Basler Vision Technologies™, Ahrensburg, Germany) operating at 60 frames per second. Imaging parameters were set at brightness 60, contrast 750, and light intensity 1000, with threshold settings of VCL > 80 µm/s, VSL > 50 µm/s, and VAP > 25 µm/s. Progressive motility was defined as spermatozoa with STR > 80%, and total motility was calculated as the sum of progressive and non-progressive motility [24]. For analysis, 3 µL aliquots of each sample were placed on pre-warmed slides, covered with a coverslip, and examined on a phase-contrast microscope (Eclipse Ci-L, Nikon, Tokyo, Japan) maintained at 37 °C. At least three fields per sample were scanned under a 10× negative phase-contrast objective, and the motility and kinematics of 600–800 spermatozoa were recorded.

#### 2.3.2. Flow Cytometry Analysis

Following thawing, sperm plasma membrane integrity, acrosome integrity, viability, and mitochondrial membrane potential were assessed using flow cytometry (Guava EasyCte™, Guava® Technologies, Hayward, CA, USA) by analyzing 10,000 spermatozoa per sample.

The proportion of live sperm with intact plasma membranes was assessed using a modified method based on Câmara et al. [25]. Samples were diluted in Tris-based solution to achieve a concentration of 50 × 10^6^ sperm/mL. To 100 µL of each sample, 0.5 µL CFDA (Cat. No: C5041; 0.46 mg/mL), 0.5 µL PI (Cat. No: 81845; 0.5 mg/mL), and 200 µL Tris solution were added. Samples were then transferred to 96-well plates for analysis. Spermatozoa exhibiting green fluorescence without red fluorescence (CFDA^+^/PI^−^) were classified as viable with intact plasma membranes.

The proportion of live and acrosome-intact spermatozoa was evaluated using FITC-conjugated peanut agglutinin (FITC-PNA, Cat. No: L7381) and propidium iodide (PI, Cat. No: 81845) following Marco-Jiménez et al. [26]. Samples were diluted to 1 × 10^6^ sperm/mL in Tris buffer, centrifuged at 700 rpm for 5 min, and resuspended in 1000 µL Tris buffer. For staining, 100 µL of each sample was mixed with 2 µL FITC-PNA (100 µg/mL), 2 µL PI (0.5 mg/mL), and 200 µL Tris buffer in the dark, incubated at room temperature for 10 min, and then transferred to 96-well plates. Samples were analyzed by flow cytometry with fluorescence emission detected at 519–590 nm.

Sperm viability was assessed using the Zombie Green™ viability kit (Cat. No: 423112, BioLegend, CA, USA). The dye was prepared by adding 100 μL of DMSO to a vial of Zombie Green provided in the kit. Prior to staining, sperm samples were washed with PBS (400× *g*, 5 min) and adjusted to a final concentration of 1 × 10^6^ sperm/mL in PBS. Each sample was then incubated with 1 μL of Zombie Green dye at room temperature in the dark for 30 min [27]. Flow cytometric analysis was performed using a 488 nm blue laser for excitation.

Sperm mitochondrial membrane potential was assessed using JC-1 dye (Cat. No: T4069). Thawed semen was diluted to 50 × 10^6^ sperm/mL in Tris-based buffer. Subsequently, 200 µL of Tris solution and 0.5 µL of JC-1 stock solution (3 mm in DMSO) were added. Samples were incubated at 38 °C for 40 min. Spermatozoa showing orange fluorescence in flow cytometry analysis were considered to have high mitochondrial membrane potential (hMMP) [28].

### 2.4. Determination of ProAKAP4 Concentration by ELISA

The goat-specific Goat 4VDX-18K9 ELISA kit (4BioDx, Lille, France) was used to measure proAKAP4 concentration in thawed semen samples (two straws per sample). The method followed the manufacturer’s instructions. Briefly, 40 µL of the kit’s lysis solution was added to 20 µL of sample and vortexed for 1 min at 3000 rpm. Subsequently, 190 µL of the kit’s dilution buffer was added. Standards (100 µL) and samples (150 µL) were added to antibody-coated microplates according to the manufacturer’s protocol. After 120 min of incubation at room temperature and washing, 100 µL of proAKAP4 detection antibody was added to each well and incubated for 60 min at room temperature. Following washing, substrate solution was added to each well, and after 10 min of incubation in the dark, the reaction was terminated with stop solution. Optical density was measured spectrophotometrically at 450 nm using a microplate reader (BioTek 800TS, Winooski, VT, USA). ProAKAP4 concentrations were expressed as ng/mL per 10^6^ spermatozoa.

### 2.5. Statistical Analyses

Statistical analyses were performed using SPSS software (version 22.0, IBM Corp., Armonk, NY, USA). Prior to selecting the appropriate statistical methods, data were assessed for normal distribution. The Kruskal–Wallis test was used to compare the mean proAKAP4 concentrations among the low, medium, and high groups. The Mann–Whitney U test was applied to compare proAKAP4 concentrations between Boer and Anglo-Nubian goat semen samples. For inter-breed comparisons, Student’s t-test was used for low and high groups, while the Mann–Whitney U test was employed for medium groups. Comparisons of spermatological parameters among the three groups were carried out using ANOVA followed by Duncan’s post hoc test. Pearson’s correlation test was conducted to evaluate the relationship between proAKAP4 concentration and spermatological parameters. Data were expressed as mean ± SE, and differences were considered statistically significant at *p* < 0.05.

## 3. Results

**Figure 1:**
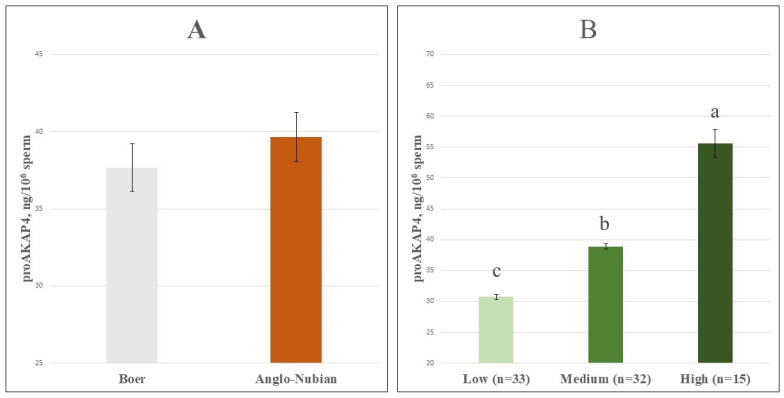
(**A**). ProAKAP4 concentrations (ng/10^6^ sperm) in post-thaw semen samples from Boer and Anglo-Nubian goats (*p* > 0.05). (**B**). Bar chart showing proAKAP4 groups. Different letters above bars indicate statistically significant differences (F = 173.467, *p* < 0.001).

A total of 80 post-thaw semen samples were analyzed, and the proAKAP4 concentrations measured by ELISA ranged from 24.42 to 77.43 ng/10^6^ sperm. The overall mean proAKAP4 concentration across all samples was 38.66 ± 1.11 ng/10^6^ sperm. The mean proAKAP4 concentration was 37.67 ± 1.54 ng/10^6^ sperm in Boer goats and 39.65 ± 1.60 ng/10^6^ sperm in Anglo-Nubian goats (Figure 1A), with no significant difference detected between breeds (*p* > 0.05).

**Figure 2:**
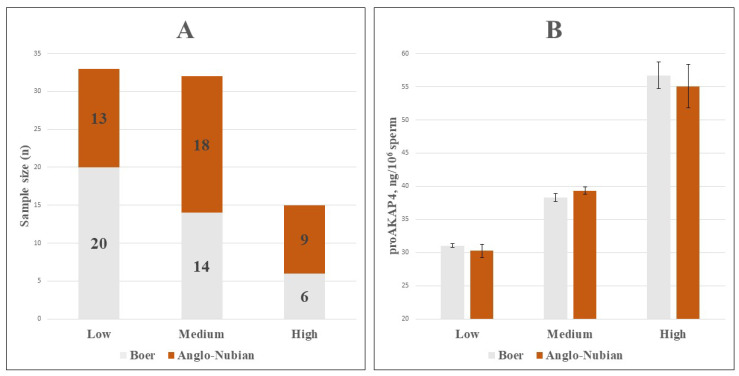
(**A**). Bar chart showing the distribution of proAKAP4 groups across breeds. (**B**). Comparison of proAKAP4 concentrations (ng/10^6^ sperm) between breeds within proAKAP4 groups (*p* > 0.05).

Based on proAKAP4 levels, samples were classified into Low (proAKAP4 < 35.00 ng/10^6^ sperm, *n* = 33), Medium (35.00 ng/10^6^ sperm ≤ proAKAP4 < 45.00 ng/10^6^ sperm, *n* = 32), and High (proAKAP4 ≥ 45.00 ng/10^6^ sperm, *n* = 15) groups, with mean values of 30.75 ± 0.41, 38.88 ± 0.41, and 55.63 ± 2.25 ng/10^6^ sperm, respectively (Figure 1B). These groups differed significantly from one another (*p* < 0.001). The distribution of proAKAP4 groups within goat breeds is presented in Figure 2A. The mean values of the proAKAP4 groups (Figure 2B) were found to be similar between breeds (*p* > 0.05).

The motility and kinematic parameters determined by CASA are presented in Table 1. Total motility (%), progressive motility (%), and BCF (Hz) increased proportionally with proAKAP4 concentration, reaching the highest values in the High group and the lowest in the Low group (*p* < 0.05). VAP (µm/s), VSL (µm/s), VCL (µm/s), and ALH (µm) values were significantly higher in the High group (*p* < 0.05), whereas no difference was observed between the Low and Medium groups (*p* > 0.05). The sperm STR (%) value was higher in the High group compared to the Low group (*p* < 0.05), while LIN (%) and WOB (%) values were similar across all groups (*p* > 0.05).

The flow cytometry findings are shown in Table 2. Acrosome integrity (%) did not differ among the groups (*p* > 0.05). However, plasma membrane integrity (%), viability (%), and hMMP (%) were significantly higher in the High proAKAP4 group compared to the Low group (*p* < 0.05).

**Figure 3:**
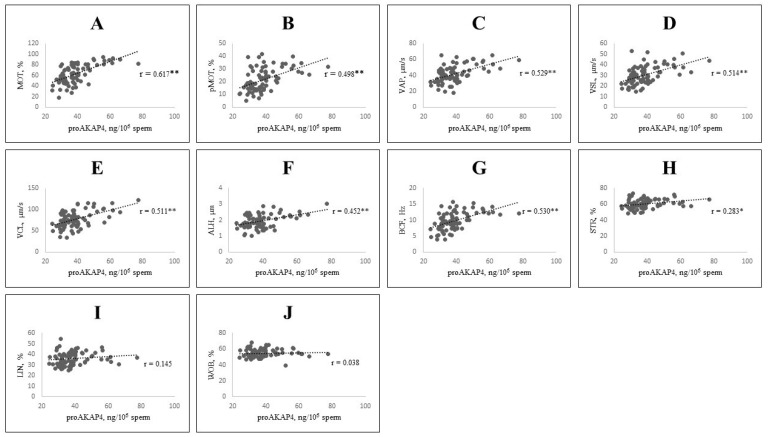
Scatter plots showing the correlation between CASA parameters and proAKAP4 concentration (* *p* < 0.05; ** *p* < 0.001). Each dot represents an individual sample, while dashed lines denote the linear regression trend. (**A**): Total motility; (**B**): Progressive motility; (**C**): VAP, average path velocity; (**D**): VSL, straight-line velocity; (**E**): VCL, curvilinear velocity; (**F**): ALH, amplitude of lateral head displacement; (**G**): BCF, beat cross frequency; (**H**): STR, straightness; (**I**): LIN, linearity; (**J**): WOB, wobble.

**Figure 4:**
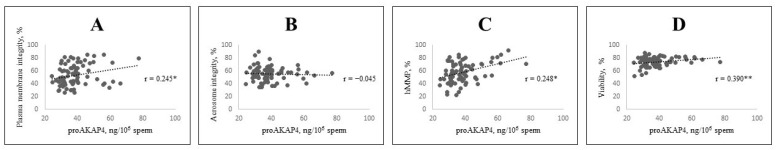
Scatter plots showing the correlation between flow cytometry parameters and proAKAP4 concentration (* *p* < 0.05; ** *p* < 0.001). Each dot represents an individual sample, while dashed lines denote the linear regression trend. (**A**): Plasma membrane integrity; (**B**): Acrosome integrity; (**C**): high mitochondrial membrane potential; (**D**): Viability.

In post-thaw semen samples, proAKAP4 levels (ng/10^6^ sperm) were significantly positively correlated with total motility (%), progressive motility (%), VAP (µm/s), VSL (µm/s), VCL (µm/s), ALH (µm), BCF (Hz), and sperm viability (%) (*p* < 0.001). Additionally, positive correlations were observed with STR (%), sperm plasma membrane integrity (%), and hMMP (%) (*p* < 0.05). No correlations were found between proAKAP4 levels and LIN (%), WOB (%), or acrosome integrity (%) (*p* > 0.05). Scatter plots illustrating the correlation coefficients (r) are presented in Figure 3 and Figure 4.

## 4. Discussion

The quality of post-thaw semen is crucial in reproductive technologies such as artificial insemination, significantly influencing fertility rates and overall reproductive success across various species. Several factors affect the quality of semen after thawing, including the composition of extenders, the freezing and thawing protocols, and the inherent characteristics of the spermatozoa. The assessment of post-thaw sperm quality is multifaceted, integrating motility, structural integrity, and biochemical markers of health. Understanding these components is vital for optimizing cryopreservation protocols and enhancing fertility outcomes across different species.

Contemporary research has increasingly emphasized the significance of molecular markers and signaling pathways in determining sperm functionality and male fertility outcomes. Among these molecular components, A-kinase anchor protein 4 (AKAP4) has emerged as a critical biomarker and functional element within mammalian spermatozoa. The mechanistic role of AKAP4-mediated phosphorylation processes has been established as fundamental to the activation of signaling cascades governing sperm motility regulation. These phosphorylation events facilitate cyclic adenosine monophosphate (cAMP)-dependent protein kinase A (PKA) activity, which serves as a central regulatory mechanism for diverse sperm functions, including hyperactivation and capacitation processes that are essential for enhanced fertilization potential [29]. The precursor form, ProAKAP4, demonstrates strategic localization within the fibrous sheath of the sperm flagellum, where it plays an integral role in maintaining optimal sperm motility and facilitating essential cellular functions required for successful fertilization events [17,30,31].

In the present study, the post-thawing sperm proAKAP4 concentration in goats was determined to be 38.66 ± 1.11 ng/10^6^ sperm. Previous studies across different species have reported considerable variation in this parameter, ranging from approximately 7 to 70 ng/10^6^ sperm [32,33,34]. The observed concentration disparities among studies can be attributed to interspecies biological variations as well as differences in the quality of semen samples utilized for measurements. Notably, our investigation revealed that proAKAP4 protein concentrations in semen samples obtained from Boer and Anglo-Nubian goat breeds did not demonstrate breed-dependent variations (*p* > 0.05). Furthermore, similar proAKAP4 levels were observed across low-, medium-, and high-concentration semen samples for both breeds examined (*p* > 0.05). The findings obtained in the present study are in line with the results reported by Dordas-Perpinyà et al. [33].

One of the most compelling indicators of successful fertilization is the progressive motility of sperm, which is crucial for navigating through the female reproductive tract to reach and fertilize the egg. Sperm that exhibit high motility tend to have greater chances of successful fertilization, as demonstrated by extensive research indicating a direct correlation between motility and fertility outcomes in assisted reproductive technologies [35]. Therefore, the evaluation of sperm motility characteristics and the identification of factors affecting these properties are of paramount importance, particularly in frozen-thawed sperm exposed to high stress factors. The present study demonstrated that post-thaw motility of goat spermatozoa was significantly influenced by proAKAP4 concentration. Samples characterized by higher proAKAP4 concentrations displayed superior total motility, progressive motility, and sperm kinetic velocity parameters (*p* < 0.05).

Spermatozoa proAKAP4 concentration has been reported to be higher in bulls with high fertility [17,31]. Additionally, positive correlations have been documented between proAKAP4 concentration and sperm progressive motility, linearity, and straightness [17,31,36]. Similarly, bulls with elevated proAKAP4 levels have been shown to exhibit higher total and progressive motility [37,38]. Furthermore, positive correlations have been reported between proAKAP4 levels and total motility, progressive motility, VSL, and VCL values obtained after 3 h of post-thaw incubation [15]. However, de Almeida et al. [34] found that X-sorted bull semen contained higher proAKAP4 concentrations compared to non-sorted samples. Interestingly, while no correlation was detected between proAKAP4 levels and sperm motility or kinematic parameters in X-sorted samples, positive correlations were observed between proAKAP4 concentration and total motility, progressive motility, and rapid sperm percentages in non-sorted samples.

The presence of proAKAP4 has also been demonstrated in stallion and donkey spermatozoa, with reports indicating that the concentration of this protein decreases over time following thawing. Furthermore, proAKAP4 levels have been reported to show positive correlations with total motility, progressive motility, and various kinematic velocity parameters of spermatozoa [16,32,33,39,40]. Similar findings have been observed in canine sperm, with proAKAP4 protein reported to have an effect on motility parameters [18,41]. However, studies conducted in cats have not observed significant correlations between CASA parameters and proAKAP4 levels; therefore, it has been recommended that proAKAP4 should not be used as a reliable indicator of sperm quality in feline semen [42].

Although there are insufficient studies on proAKAP4 in small ruminants, research conducted in rams has demonstrated positive correlations between proAKAP4 concentration and total and progressive motility. However, cryopreservation procedures have been reported to negatively affect proAKAP4 levels, leading to a reduction in its concentration [19]. Additionally, in a study based on 36 ejaculates from 18 goats (Alpine and Saanen), proAKAP4 levels were reported to show positive correlations with sperm motility parameters (parameter type not reported) and fertility [20].

Flow cytometry has emerged as a pivotal tool in the assessment of semen quality, providing a multiparametric analysis that enhances the understanding of sperm functionality and viability. This technique allows for rapid evaluation of various sperm parameters that are critical to determining fertility potential across species. Sperm plasma membrane integrity is critically associated with fertilization success, serving as an essential parameter for evaluating sperm quality across species. The integrity of the sperm plasma membrane is crucial for a range of physiological processes, including capacitation, acrosomal reaction, and ultimately, the fusion of sperm and oocyte membranes, which are critical for successful fertilization [43]. The viability of sperm is a direct indicator of its potential for successful fertilization, making sperm assessments integral to reproductive technology. Various studies have highlighted that maintaining high levels of sperm viability pre- and post-cryopreservation is critical for ensuring that sperm retains its fertilization capacity. Research indicates that current freezing and thawing protocols can result in significant losses of sperm viability, often exceeding 30% [7]. In the present study, flow cytometry was employed to assess sperm plasma membrane integrity, acrosome integrity, viability, and mitochondrial membrane potential. The relationships between these parameters and goat sperm proAKAP4 concentration were investigated and reported for the first time, thereby contributing novel findings to the existing literature. It was determined that sperm from goats with high proAKAP4 concentrations exhibited higher plasma membrane integrity and viability rates compared to the group with low concentrations (*p* < 0.05). The findings of our study are supported by numerous previous investigations. Bastan and Akçay [15] demonstrated a negative correlation between tail defects and proAKAP4 concentration in bull sperm. Kudratullah et al. [38] reported that sperm samples with high viability rates also exhibited elevated proAKAP4 concentrations. de Almeida et al. [34] showed a positive correlation between sperm plasma membrane integrity and proAKAP4 concentration in conventional bull semen samples. Pardede et al. [17] reported that bulls with high fertility rates had significantly higher proAKAP4 levels and demonstrated strong positive correlations between proAKAP4 concentration and sperm plasma membrane integrity. These researchers suggested that proAKAP4 levels in bulls could serve as an important biomarker for potential fertilization success. Similarly, Riesco et al. [19] revealed positive correlations between proAKAP4 protein levels and viability and negative correlations with apoptosis in rams.

Although ProAKAP4 protein is predominantly reported to localize in the principal piece of the sperm tail, recent evidence has also revealed its presence in the midpiece, sperm head, and acrosomal regions [17]. Previous literature has documented correlations between proAKAP4 protein and intact acrosome presence across different species, suggesting that abundance of proAKAP4 protein in spermatozoa may be associated with enhanced acrosomal integrity and non-capacitated spermatozoa status [17,33]. However, the present study represents the first investigation of the relationship between acrosomal integrity and proAKAP4 concentration in goats, revealing no significant correlation between these parameters. This finding challenges the previously established associations and suggests that the relationship between proAKAP4 and acrosomal function may exhibit species-specific variations.

The mitochondrial membrane potential (MMP) serves as an important indicator of mitochondrial function. Studies have shown that higher MMP values correlate with improved sperm motility, viability, and overall fertilization capacity [44]. MMP not only reflects energy availability but also influences the sperm’s ability to undergo capacitation. Exploratory studies have illuminated the vital role of MMP in sperm capacitation, a prerequisite for successful fertilization. Capacitation is associated with an increase in mitochondrial activity, which must be sustained for sperm to achieve hyperactivation—a key component for penetrating the oocyte’s zona pellucida [45]. In the present study, a positive correlation was observed between mitochondrial membrane potential and proAKAP4 protein levels (*p* < 0.05), with spermatozoa possessing high proAKAP4 concentrations demonstrating higher mitochondrial membrane potential compared to those with low concentrations (*p* < 0.05). Our findings are consistent with the study by Pardede et al. [17] in bulls. However, they are inconsistent with the results reported by Dordas-Perpinya et al. [40] in donkeys. In that investigation, sperm mitochondrial membrane potential was similarly examined using JC-1 staining, but no significant correlation with proAKAP4 levels was reported. The researchers explained this phenomenon by suggesting that there may be a certain independence between energy production and motility transmission in spermatozoa. The discrepancy between studies may be attributed to interspecies biological differences. On the other hand, spermatozoa are highly specialized cells whose compartmentalized functions are largely regulated by cAMP signaling, with AKAPs orchestrating compartment-specific activities [46]. cAMP-dependent PKARII is enriched in cytoplasmic and tail regions, particularly within the fibrous sheath and mitochondria [47,48]. Therefore, the roles of proAKAP4 and AKAP4 in stabilizing mitochondrial function are particularly important and require detailed investigation.

## 5. Conclusions

In conclusion, the findings obtained demonstrate that the proAKAP4 protein can serve as a potential biomarker for determining post-thaw semen quality in goats. Specifically, proAKAP4 concentrations have been established to correlate with sperm motility parameters and with viability, plasma membrane integrity, and mitochondrial function. This investigation confirms that proAKAP4 represents a reliable indicator of post-thaw semen quality in both Boer and Anglo-Nubian goat breeds while emphasizing the importance of conducting additional studies across different breeds to further validate these findings and establish broader applicability.

## Figures and Tables

**Table 1:** CASA parameter values (mean ± SE) in Low, Medium, and High proAKAP4 groups.

CASA Parameters	proAKAP4 Groups				
	Low (n = 33)	Medium (n = 32)	High (n = 15)	F	*p*-Value
MOT, %	53.08 ± 2.44 ^c^	63.05 ± 2.74 ^b^	81.86 ± 3.32 ^a^	20.442	<0.001
pMOT, %	17.73 ± 1.33 ^c^	22.31 ± 1.55 ^b^	29.14 ± 1.50 ^a^	11.036	<0.001
VAP, µm/s	38.30 ± 1.60 ^b^	40.86 ± 1.73 ^b^	52.90 ± 2.05 ^a^	13.326	<0.001
VSL, µm/s	26.97 ± 1.37 ^b^	30.29 ± 1.43 ^b^	38.33 ± 1.55 ^a^	11.282	<0.001
VCL, µm/s	70.67 ± 2.94 ^b^	76.44 ± 3.07 ^b^	95.49 ± 4.74 ^a^	10.662	<0.001
ALH, µm	1.82 ± 0.06 ^b^	1.89 ± 0.06 ^b^	2.28 ± 0.10 ^a^	7.334	0.001
BCF, Hz	8.43 ± 0.43 ^c^	9.94 ± 0.46 ^b^	12.45 ± 0.38 ^a^	14.441	<0.001
STR, %	58.72 ± 1.03 ^b^	60.98 ± 1.00 ^ab^	62.83 ± 1.18 ^a^	3.060	0.053
LIN, %	34.85 ± 1.20	36.66 ± 1.06	37.38 ± 1.21	1.110	0.335
WOB, %	54.00 ± 0.96	55.38 ± 0.87	54.57 ± 1.38	0.563	0.572

Different letters within rows indicate statistically significant differences. Low, proAKAP4 < 35.00 ng/10^6^ sperm; Medium, 35.00 ng/10^6^ sperm ≤ proAKAP4 < 45.00 ng/10^6^ sperm; High, proAKAP4 ≥ 45.00 ng/10^6^ sperm. F, the test statistic representing the ratio of between-group to within-group variance in ANOVA. CASA, computer-assisted sperm analysis; MOT, total motility; pMOT, progressive motility; VAP, average path velocity; VSL, straight-line velocity; VCL, curvilinear velocity; ALH, amplitude of lateral head displacement; BCF, beat cross frequency; STR, straightness; LIN, linearity; WOB, wobble.

**Table 2:** Flow cytometric sperm analysis findings (mean ± SE) in Low-, Medium-, and High-proAKAP4 groups.

Flow Cytometry Parameters	proAKAP4 Groups				
	Low (n = 33)	Medium (n = 32)	High (n = 15)	F	*p*-Value
Membrane integrity, %	48.74 ± 2.50 ^b^	53.78 ± 2.86 ^ab^	60.09 ± 4.93 ^a^	2.656	0.077
Acrosome integrity, %	55.28 ± 2.29	54.11 ± 1.78	54.72 ± 2.17	0.087	0.916
Viability, %	72.41 ± 1.54 ^b^	74.14 ± 1.05 ^ab^	77.14 ± 0.94 ^a^	3.741	0.028
hMMP, %	52.93 ± 2.70 ^b^	59.11 ± 2.52 ^ab^	65.47 ± 4.16 ^a^	2.343	0.103

Different letters within rows indicate statistically significant differences. F, the test statistic representing the ratio of between-group to within-group variance in ANOVA. Low, proAKAP4 < 35.00 ng/10^6^ sperm; Medium, 35.00 ng/10^6^ sperm ≤ proAKAP4 < 45.00 ng/10^6^ sperm; High, proAKAP4 ≥ 45.00 ng/10^6^ sperm. hMMP, high mitochondrial membrane potential.

## Data Availability

The original contributions presented in this study are included in the article. Further inquiries can be directed to the corresponding author.

## Abbreviations

The following abbreviations are used in this manuscript:

AKAP4	A-kinase anchor protein 4
proAKAP4	Precursor a-kinase anchor protein 4
DNA	Deoxyribonucleic acid
CASA	Computer-assisted sperm analysis
ELISA	Enzyme-linked immunosorbent assay
MOT	Total motility
PMOT	Progressive motility
VAP	Average path velocity
VSL	Straight-line velocity
VCL	Curvilinear velocity
ALH	Amplitude of lateral head displacement
STR	Straight-line velocity
LIN	Linearity
WOB	Wobble
CFDA	Carboxyfluorescein diacetate
PI	Propidium iodide
PNA	Peanut agglutinin
DMSO	Dimethyl sulfoxide
JC-1	1,1’,3,3’-Tetraethyl-5,5’,6,6’-tetrachloroimidacarbocyanine iodide
SPSS	Statistical package for the social sciences
ANOVA	Analysis of variance
HMMP	High mitochondrial membrane potential
MMP	Mitochondrial membrane potential
PKA	Protein kinase A
CAMP	Cyclic adenosine monophosphate
